# Intravital Imaging of Pulmonary Immune Response in Inflammation and Infection

**DOI:** 10.3389/fcell.2020.620471

**Published:** 2021-01-15

**Authors:** Nazli Alizadeh-Tabrizi, Stefan Hall, Christian Lehmann

**Affiliations:** ^1^Department of Physiology & Biophysics, Dalhousie University, Halifax, NS, Canada; ^2^Department of Anesthesia, Pain Management and Perioperative Medicine, Dalhousie University, Halifax, NS, Canada

**Keywords:** inflammation, infection, lung, intravital microscopy, leukocytes

## Abstract

Intravital microscopy (IVM) is a unique imaging method providing insights in cellular functions and interactions in real-time, without the need for tissue extraction from the body. IVM of the lungs has specific challenges such as restricted organ accessibility, respiratory movements, and limited penetration depth. Various surgical approaches and microscopic setups have been adapted in order to overcome these challenges. Among others, these include the development of suction stabilized lung windows and the use of more advanced optical techniques. Consequently, lung IVM has uncovered mechanisms of leukocyte recruitment and function in several models of pulmonary inflammation and infection. This review focuses on bacterial pneumonia, aspiration pneumonia, sepsis-induced acute lung Injury, and cystic fibrosis, as examples of lung inflammation and infection. In addition, critical details of intravital imaging techniques of the lungs are discussed.

## Introduction

The application of microscopy to live tissues (syn.: intravital microscopy, IVM), allows imaging of cellular processes at high resolution in real-time. This technology provides unique information in addition to *ex vivo/in vitro* methods without the need for removing the tissue from the physiological environment, processing sections, fixation, or staining (Wells et al., 2018). Various experimental IVM models have been successfully established to demonstrate time-sequential cellular changes in several organs under different physiological and pathophysiological conditions, e.g., in liver, brain, or skeletal muscle (Kuhnle et al., 1993; Kramer et al., 2000; McCormack et al., 2000; Mempel et al., 2003; Khandoga et al., 2005; Kuebler et al., 2007; Lindert et al., 2007; Tabuchi et al., 2008; Ochi et al., 2019). Yet, one of the most difficult organs for intravital imaging is the lung due to limited accessibility based on its enclosed position within the body, its respiratory movements, and the physical impact of the heart beat (Tabuchi et al., 2008). Although there are different imaging strategies to study cell populations—both, *in vitro* such as histology, immunohistochemistry, and *in vivo* such as MRI, and CT–any of these methods is not able to visualize dynamic cellular behavior and mechanisms during physiological and pathophysiological condition. For instance, clinical modalities such as positron emission tomography (PET), magnetic resonance imaging (MRI), and computed tomography (CT) can provide non-invasive images of the lungs. However, they lack the resolution necessary to study cellular mechanisms. Classical fluorescence techniques using ultraviolet (UV) light marked the beginning of lung IVM and are still used by the scientific community (Entenberg et al., 2015; Fiole and Tournier, 2016). Confocal microscopy and modern fluorescence-based technologies, such as multiphoton excitation microscopy have revealed more complex as well as deeper pulmonary structures (Krahl, 1963; Looney et al., 2011; Presson et al., 2011; Entenberg et al., 2015). Ultimately, all these achievements largely contributed to the establishment of intravital imaging as a gold standard in cellular lung research (Fiole and Tournier, 2016; Rodriguez-Tirado et al., 2016). In this review, we first describe the technical aspects of lung IVM. In the second part, we review experimental lung inflammation and infection models utilizing lung IVM.

## Setup

Lung IVM can be performed by using different microscopic techniques. Historically, fluorescence microscopy was used first to study pulmonary inflammation and infection. Newer technologies include laser scanning confocal microscopy, single-photon microscopy, and two/multiphoton microscopy (Fiole and Tournier, 2016; Wang, 2016).

### Fluorescence Microscopy

Fluorescence microscopy is a technique in which a sample stained with fluorescent dye is visualized using a halogen lamp such as xenon, mercury, or tungsten as a light source (Lindon et al., 2016). The objective lens focuses the excitation light, allowing maximal collection of emitted fluorescence and magnification for the observation of fine details (Herman, 1998). The fluorescent dye in the sample is excited with a relatively short wavelength, usually blue or ultraviolet light, that matches the fluorophore excitation wavelength. The emitted fluorescence has longer wavelength and less energy in comparison to the absorbed excitation light, which is blocked by the emission filter. A beam splitter is required to separate the excitation light from emission fluorescence and prevent overlap in their light paths. The dichroic mirror reflects the shorter wavelength excitation light and transmits the longer wavelength of emitted fluorescence through the barrier filter (Lichtman and Conchello, 2005). Emitted fluorescence from the specimen that is collected by the objective passes through the dichroic mirror and the barrier filter to the eyepieces or detector (Sanderson et al., 2014). The selectivity for specific wavelengths facilitates the visualization of different fluorescent objects as a bright structure against a dark background (Lichtman and Conchello, 2005; Lindon et al., 2016). Although fluorescence microscopy has been widely used to study tissues *in vivo* (Lindon et al., 2016), its application in lung IVM is limited to superficial layers and low resolution (Witte, 1992; Presson et al., 2011; Lefrançais et al., 2017).

### Confocal Microscopy

Confocal microscopes differ from conventional fluorescence microscopes in that they use a laser light source and improve image resolution due to reduced detection of out of focus light (Norman, 2005). According to the principle of confocal microscopy, light is collected through narrow apertures (pinholes) that exclude out of focus light. The elimination of out of focus light results in an improvement in lateral and axial resolution. Both laser scanning and spinning disk confocal microscopes pass a single beam of laser light through the pinhole. It should be noted that while confocal laser scanning microscopy focuses the light through one small pinhole in order to sequentially scan the sample point by point, confocal spinning disk microscopy exploits multiple pinholes for simultaneous confocal illumination (Masedunskas et al., 2012; Sanderson et al., 2014; Lefrançais et al., 2017). Therefore, either X–Y-deflection of the laser or a spinning disk with a spatial array of pinholes and automated-focus (z-axis) control enables the visualization of sequential optical sections of the specimen and three-dimensional images. Several factors determine the resolution of confocal microscopy, including the diameter of the pinhole, the light wavelength and the numerical aperture of the objective (Tauer, 2002; Norman, 2005). Confocal microscopy has been used to visualize capillary and alveolar networks in the lung. Although confocal microscopy provides superior spatial resolution, live imaging with this microscope results in a large proportion of emitted light being blocked due to the small size of the pinhole. In addition, penetration depth in confocal laser scanning microscopy is limited to about 50–100 μm which precludes imaging of deeper tissues. Moreover, imaging with confocal microscopy requires a bright sample in order to negate the need for a strong excitation signal which may otherwise cause photodamage and photobleaching. In this regard, spinning disk confocal microscopy is superior as its high acquisition speed helps to restrict photobleaching (Croix et al., 2006; Masedunskas et al., 2012; Lefrançais et al., 2017).

### Multiphoton Microscopy

Multiphoton microscopy involves application of an infrared laser light source and subsequent absorption of lower energy photons by a fluorophore inside the tissue (Tauer, 2002; Norman, 2005; Presson et al., 2011; Lefrançais et al., 2017). This method uses long-wavelength photons to penetrate farther into tissues and allow for imaging of thicker sections (Lefrançais et al., 2017). Using multiphoton microscopy, lungs have been imaged to a depth of ~500 μm (Croix et al., 2006). Hence, application of multiphoton microscopy is superior in intravital studies (Norman, 2005). However, multiphoton microscopy also has some drawbacks. Although the likelihood of phototoxicity is decreased in comparison to conventional fluorescence and confocal microscopy, photodamage is still considered as a disadvantage for multiphoton microscopy when compared to single-photon microscopy, at least in the focal plane (Tauer, 2002; Croix et al., 2006; Presson et al., 2011). Additionally, increased penetration depth leads to significant decrease in spatial resolution (Niesner et al., 2007). Since excitation only occurs at the focal point and multiphoton excitation efficiency of fluorophores are very low in comparison to single photon, longer acquisition times are required. This has the effect of restricting observation of dynamic processes which possess high temporal resolution (Niesner et al., 2007; Presson et al., 2011). Despite these drawbacks, multiphoton microscopy is nonetheless advantageous for IVM studies because the infrared excitation light is less prone to scatter, which allows for deeper penetration into the tissue (Croix et al., 2006; Lefrançais et al., 2017).

### Surgery

The surgical preparation needed for intravital lung imaging requires that the animals are anesthetized, usually by intraperitoneal injection or inhalation of an anesthetic drug. Animals will be intubated or tracheotomized to facilitate mechanical ventilation (Fiole and Tournier, 2016). The following steps include techniques to create a thoracic window and mechanically stabilize the lung in order to perform IVM. Several techniques have been developed in recent decades. In 1926, Wearn et al. (1926) described for the first time a surgical procedure in dogs, where the thoracic wall was resected to the pleural layer. A second opening was made through the diaphragm down to the pleura for illumination (Rodriguez-Tirado et al., 2016). Other authors studied the physiological movement of the canine lung via an implanted lung window over a relatively stationary region (Wagner, 1965), or utilized a vacuum during the surgical window preparation to stabilize the tissue (Wagner, 1969). In general, two alternative lung stabilization methods for imaging in mice are used today, namely gluing of the parenchyma onto a glass coverslip (Kreisel et al., 2010) or the utilization of a suction system to stabilize the lung under a glass window (Looney et al., 2011). Other techniques have their own merits and pitfalls, and no one has excelled in comparison to another (Fiole and Tournier, 2016). For example, bronchus clamping, and sequential apnea impact the normal gas exchange in the lung and may cause atelectasis, which refers to impaired gas exchange resulting from reversible collapse of small airways (Grott and Dunlap, 2020). Gated imaging and oversampled acquisition do not have these disadvantages but require high-speed or specialized imaging equipment, which is not widely accessible. Neither gluing of the lung or utilization of the suction window cause the above-mentioned drawbacks, but either may result in shear force induced injury. In recent years, the suction window has been miniaturized and adapted for use in mice (Rodriguez-Tirado et al., 2016). Confocal and multiphoton microscopy have been used (Funakoshi et al., 2000; Looney et al., 2011; Presson et al., 2011) to obtain excellent high-resolution imaging (Entenberg et al., 2015). At the end of the experiment, mice are always euthanized in accordance with international animal care guidelines (Fiole and Tournier, 2016).

### Cell Labeling

With recent advances of fluorescent probes, fluorescent proteins and exogenous fluorophores, IVM allows the detection of molecular events with subcellular resolution in real time in the intact animal (Presson et al., 2011). Tracking the movement, morphology and behavior of leukocytes and blood vessels in the lung requires labeling methods such as the use of fluorescent dyes, transgenic mice, or fluorescently stained antibodies (Kim et al., 2019). Moreover, knockout mice can be used to study the cellular mechanisms involved in pulmonary pathophysiological conditions (Aird, 2003; Gill et al., 2015) (Table 1).

**Table 1:** Cell labeling strategies that have been used for lung IVM.

**Florescent probes/dye**	**Florescent antibodies**	**Florescent reporter mice**	**Knockout Mice**
**Leukocytes** • Rhodamine 6G (Razavi et al., 2004; Kreisel et al., 2010; Roller et al., 2013; Brown et al., 2014; Gill et al., 2014, 2015) **Microvessels** • Fluorescein isothiocyanate (FITC)-dextran (Tabuchi et al., 2008; Brown et al., 2014) • FITC-albumin (Noda et al., 2014) • Texas Red dextran (Alves et al., 2018) **Cell death** • Propidium iodide (PI) (Gill et al., 2014, 2015)	**Neutrophil** • Ly6G (Lien et al., 1987; Kuebler et al., 1994; Lee et al., 2013) • Anti-CD45 (Naumenko et al., 2018) **Monocyte/macrophages** • Ly6C/F4/80 (Wynn et al., 2013) **Neutrophil Elastase (NE)** • Anti-neutrophil elastase (NE)-AF647 (Carestia et al., 2019) **Vascular endothelium** • Anti-CD31 (Yipp et al., 2017)	**Neutrophils** • Lysozyme M-green fluorescent protein (LysM-GFP) mice (Faust et al., 2000; Orthgiess et al., 2016) • LysM-GFP mice (Kreisel et al., 2010) **Monocyte/macrophages** • CX3CR1-GFP mice (Medina-Contreras et al., 2011; Garcia et al., 2013)	iNOS^−/−^ mice (Razavi et al., 2004) CD11b^−/−^ mice (Yipp et al., 2017) TLR4^−/−^ mice (Yipp et al., 2017) Myd88^−/−^ mice (Yipp et al., 2017)

Fluorescent dyes such as Alexa Fluor 488, FITC, phycoerythrin, and Rhodamine 6G allow for visualization of separate cells (Chiang et al., 2007). In addition, genetically different mouse strains have been utilized to visualize a specific cell subset. For instance, Lysozyme M-green fluorescent protein (LysM-GFP) mice are used for visualization of neutrophils because Lysozyme M, encoded by the *Lyz2* gene, is expressed mainly in neutrophils and partly in macrophages (Faust et al., 2000; Orthgiess et al., 2016). Kreisel et al. (2010) examined leukocyte trafficking in LysM-GFP mice (Faust et al., 2000) in which endogenous neutrophils are brightly labeled and monocytes and macrophages are labeled to a lesser extent (Chtanova et al., 2008). Another transgenic mouse line, CX3Cr1-GFP, is used for fluorescent imaging of monocytes and macrophages (CX3C chemokine receptor 1 (CX3Cr1) is a marker for macrophages and monocytes) (Medina-Contreras et al., 2011; Garcia et al., 2013).

Another method for imaging of leukocyte subsets, besides the use of fluorescent dyes or transgenic mouse lines, is the application of fluorescently stained antibodies capable of binding to specific antigens. For instance, one of the cell surface proteins that is highly expressed in murine neutrophils is Ly6G (Lee et al., 2013), whereas Ly6C and F4/80 are highly expressed in macrophages or monocytes (Wynn et al., 2013). Kuebler et al. fluorescently labeled leukocytes *in vivo* with Rhodamine 6G and imaged the subpleural microcirculation in rabbits (Kuebler et al., 1994). Lien et al. labeled neutrophils *in vitro* and used fluorescence videomicroscopy in dogs to image neutrophil in pulmonary arteries (Lien et al., 1987). These two studies demonstrated that although rolling leukocytes were observed in arteries, venules, and capillaries, the anatomical site of neutrophil margination is in the pulmonary capillaries (Lien et al., 1987; Kuebler et al., 1994). This differs from murine systemic circulation, where rolling leukocytes are predominately observed in postcapillary venules and rarely in arterioles (Broide et al., 1998). To visualize the pulmonary vasculature and determine whether neutrophils were extravascular, Kreisel et al. injected quantum-dots, and reported that neutrophils were sequestered in the pulmonary microcirculation (Lien et al., 1987; Kreisel et al., 2010). Additionally, to study cellular mechanisms for neutrophil recruitments in lung after induction of experimental sepsis, CD11b^−/−^,,TLR4^−/−^, Myd88^−/−^mice (Yipp et al., 2017) and iNOS^−/−^ mice were used (Razavi et al., 2004).

Changes in microvessel diameter and blood flow under various physiological and pathophysiological conditions is widely visualized via intravenous administration of fluorophore-conjugated dextran or albumin (Kim et al., 2019). Higher molecular weight dextran (70 kDa) is particularly useful for visualization of microvessels because extravasation through the intact endothelium is minimal. Tabuchi et al. demonstrated changes in pulmonary vessel diameters by injection of FITC-dextran into the jugular vein of mice (Tabuchi et al., 2008). Another study involved administration of intravenous FITC-albumin to enable measurement of microvascular leakage in rat mesentery through visualization of blood vessels (Alves et al., 2018)In mice with GFP-labeled immune cell populations, blood vessels have been marked with Texas Red dextran for better contrast (Noda et al., 2014). Visualization of blood vessels and immune cells can be improved through application of novel labeling materials, proper combination of inflammation models, superior surgical methods, and advances in microscopic imaging.

## Infection and Inflammation Models

### Bacterial Pneumonia

Two common nosocomial pathogens that lead to bacterial infection of the respiratory tract are *Pseudomonas aeruginosa* and *Staphylococcus aureus*. *Pseudomonas aeruginosa* is a Gram-negative opportunistic bacterium that can cause lung infection in patients with impaired immunity and is the main cause of morbidity and mortality in cystic fibrosis (CF) patients (Moradali et al., 2017). Wild type (PAO1) and human-derived (PA14, LESB58) strains are often used in animal infection models. It should be noted that while PAO1 and PA14 are localized in alveolar regions 7 days post infection, LESB58 has been shown to persist in the bronchial lumen (Kukavica-Ibrulj et al., 2008). *Staphylococcus aureus* spp. are Gram-positive bacteria and their most studied pathogenicity factors recognized by immune cells are lipoteichoic acid and peptidoglycan (Fournier and Philpott, 2005). In this context, antibiotic-resistant strains (e.g., methicillin-resistant *S. aureus*, MRSA), are commonly used for animal infection studies (Kim et al., 2014). Lung IVM can be utilized to visualize pathogen recognition and bacterial clearance by immune cells (Fournier and Philpott, 2005; Lavoie et al., 2011). In addition, dynamic immune cell responses such as formation of neutrophil clusters and neutrophil extracellular traps (NETs) are often observed in the lungs in response to bacterial infection (Lien et al., 1987; Looney and Bhattacharya, 2014; Lefrançais et al., 2018).

**Figure 1:**
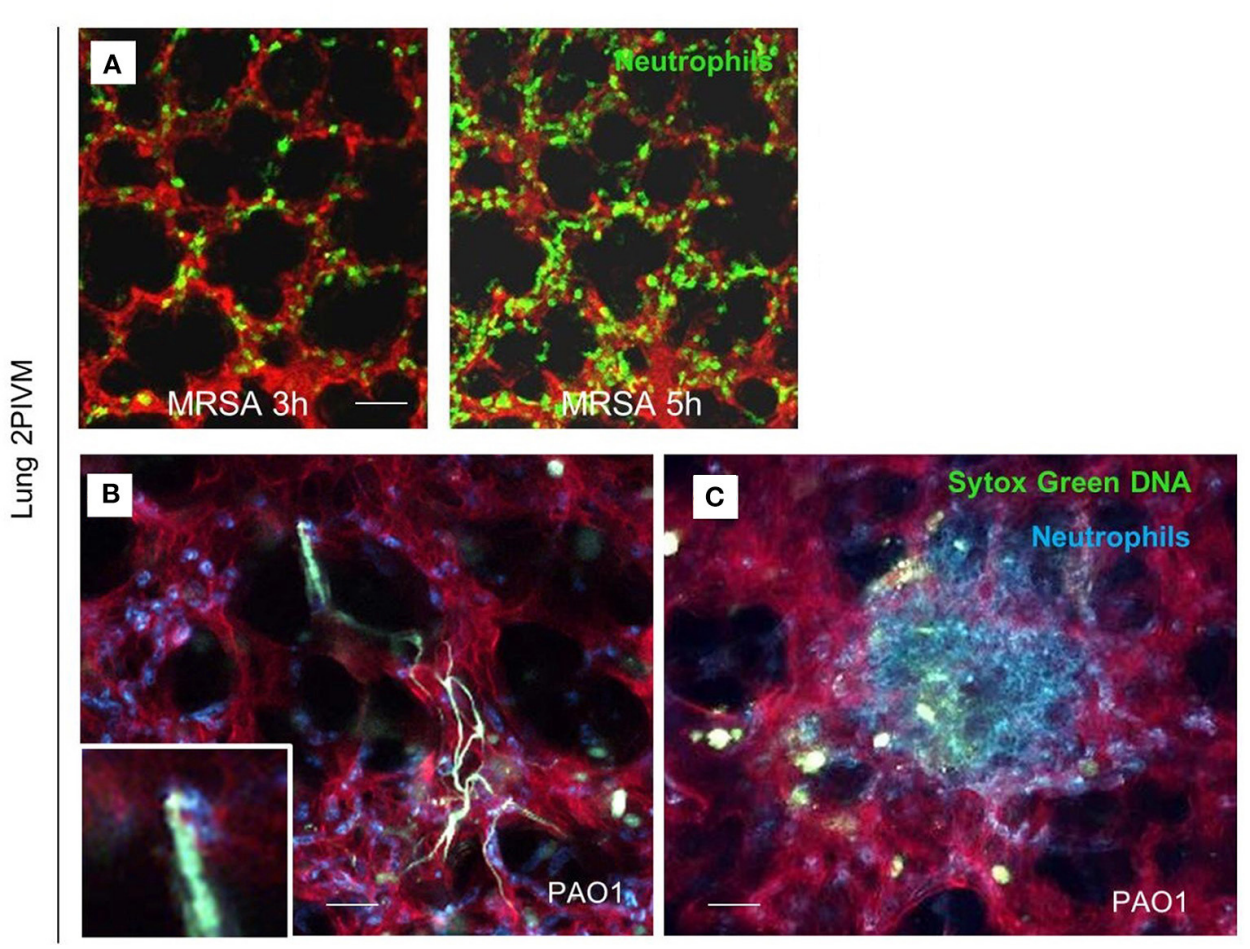
Lung 2-photon intravital microscopy. **(A)** LysM-GFP mice (green neutrophils) were challenged with MRSA (2 × 10^7^ CFU, i.t.), injected with Texas Red–dextran i.v. to stain the vasculature, and observed from 3 to 5 h after infection. **(B,C)** MRP8-mTmG mice (red vasculature, blue neutrophils) were challenged with PAO1 (5 × 10^6^ CFU, i.t.) and observed from 3 to 5 h after the infection. Extracellular DNA was stained with SYTOX Green (Lefrançais et al., 2018).

Lefrançais et al. utilized multiphoton microscopy for visualization of neutrophil recruitment and NET formation in experimental MRSA-induced pneumonia (Lefrançais et al., 2018). The left lung of the mice was exposed surgically and stabilized via a flanged thoracic window with a coverslip and vacuum suction. Infection with MRSA rapidly induced neutrophil recruitment and sequestration in the lung beginning 2 h post infection. In addition, after PAO1 challenge, neutrophil swarming and formation of neutrophils clusters around the bacteria were observed (Figure 1).

Kreisel et al. exposed the left rodent lung by thoracotomy and attach it to the cover glass using tissue glue. Non-targeted Q-dots were injected intravenously to image blood vessels, and LysM-GFP mice were used for visualization of neutrophils by means of multiphoton microscopy. Diluted *Escherichia coli* (K-12 strain) Bioparticles conjugated with tetramethylrhodamine or *L. monocytogenes* in PBS were administered intratracheally prior to imaging. Ten minutes following administration, a dramatic influx of cells from the circulation and a significant increase in resident neutrophil motility was observed in lung tissue. However, neutrophil recruitment to the lung, specifically at the transendothelial migration step, was inhibited after the depletion of blood monocytes, which suggests interaction of monocytes and neutrophils in lung inflammation (Kreisel et al., 2010).

In another study, Fiole et al. performed lung IVM via multiphoton microscopy to examine early steps of pulmonary infection by *Bacillus anthracis*, the causative agent of anthrax that impacts livestock and humans. The authors observed interactions among macrophages, DCs and spores which was considered to result in an exchange of information including exchange of pathogen-derived particles or exosomes containing pathogen-derived antigens released by macrophages directed to DCs. Results from this study indicates that infection induced by *B. anthracis* spores significantly increases long-duration (> 30 min) contact between macrophages and CX3CR1-DCs (Fiole et al., 2014). Alveolar macrophages, the most efficient phagocytes in the lung, capture spores within minutes in a first step. After 30 min, spores are transported to lymph nodes following capture by DCs (Cleret et al., 2007). However, mean cell velocity in both macrophages and CX3CR1 cells did not significantly increase correlated to increased contact ratio at 5 h post-infection (Fiole et al., 2014). Fiole et al. observed the last phase of spore transfer from macrophage to CX3CR1 cell *in situ* and *in vivo*, a mechanism previously only demonstrated *in vitro* (Blank et al., 2011).

Administration of lipopolysaccharide (LPS), a cell wall component of Gram-negative bacteria, induces an inflammatory response by activation of immune cells through Toll-like receptor (TLR)-4 (Aderem and Ulevitch, 2000; Opal, 2010; Kim et al., 2019). Following LPS administration as well as bacterial infection, neutrophils are the first group of leukocytes that respond to inflammation. This response involves adhesion and migration across the endothelium from bloodstream into inflammatory tissues. Carestia et al. studied in LPS induced inflammation the establishment of intravascular neutrophil extracellular traps (NETs) and immunothrombi by intravital microscopy. In this study, the left side of the chest was opened to access the lung in tracheotomized mice. The lung was gently immobilized with a thoracic suction attached to the manipulator on the microscope stage. The authors studied platelet aggregates, neutrophil numbers, bacterial capture by each cell type, and the number of stationary (≥30s in the same location) bacteria in contact with platelet aggregates. They visualized neutrophils through labeling with anti-Ly6G-BV42. Anti-neutrophil elastase (NE)-AF647 antibodies, which bind to extracellular neutrophil elastase were injected via tail vein, prior to IVM imaging. The authors found that platelet aggregation, neutrophil recruitment, and NET release were induced 4 h following intraperitoneal injection of LPS (Carestia et al., 2019). Pulmonary inflammation induced by inhalation of LPS from *Salmonella enteritis* (Reutershan et al., 2005) was confirmed by lung IVM with application of a mild vacuum to hold the lung under the window of a custom-built fixation device. Fluorescein isothiocyanate (FITC)- dextran (150 kDa) was used to assess the glycocalyx. The results from this study indicated that glycocalyx thickness in the lung was significantly reduced after 8 and 24 h following LPS inhalation, thus resulting in increased vascular permeability (Margraf et al., 2018).

### Viral Infections

In addition to bacterial infections, also viral infection can be studied by IVM. In particular, influenza has been studied widely in lungs using IVM. In a study from Paul Kubes group in Calgary, anesthetised, thoracotomized mice were given anti-Ly6G/GR1 and anti-CD31 to identify neutrophils and vasculature, respectively, and studied using confocal intravital microscopy. The IVM images demonstrated that after 30 min and even 2 h post infection AMs actively detected and crawled toward inhaled *P. aeruginosa* via chemotaxis. AMs crawling behavior was defected during infection with Influenza A compared to control and after infection with *P. aeruginosa* or *S. aureus*, fewer AMs from flu-infected mice captured the inhaled *P. aeruginosa* and *S. aureus*. These findings suggest that Influenza A impairs the ability of AMs to crawl and capture the inhaled bacteria and increase neutrophil infiltration (Neupane et al., 2020). In another study with similar microscopic and surgical preparation, confocal microscopy of the lung demonstrated interactions between leukocytes which were visualized using anti-CD45 and oncolytic vesicular stomatitis virus (VSV) which was identified by Alexa Fluor 647 staining. Occasional capture of virions by leukocytes within the lung vasculature was observed 10 min after i.v. injection of VSV-AF647 (Naumenko et al., 2018).

### Cystic Fibrosis

**Figure 2:**
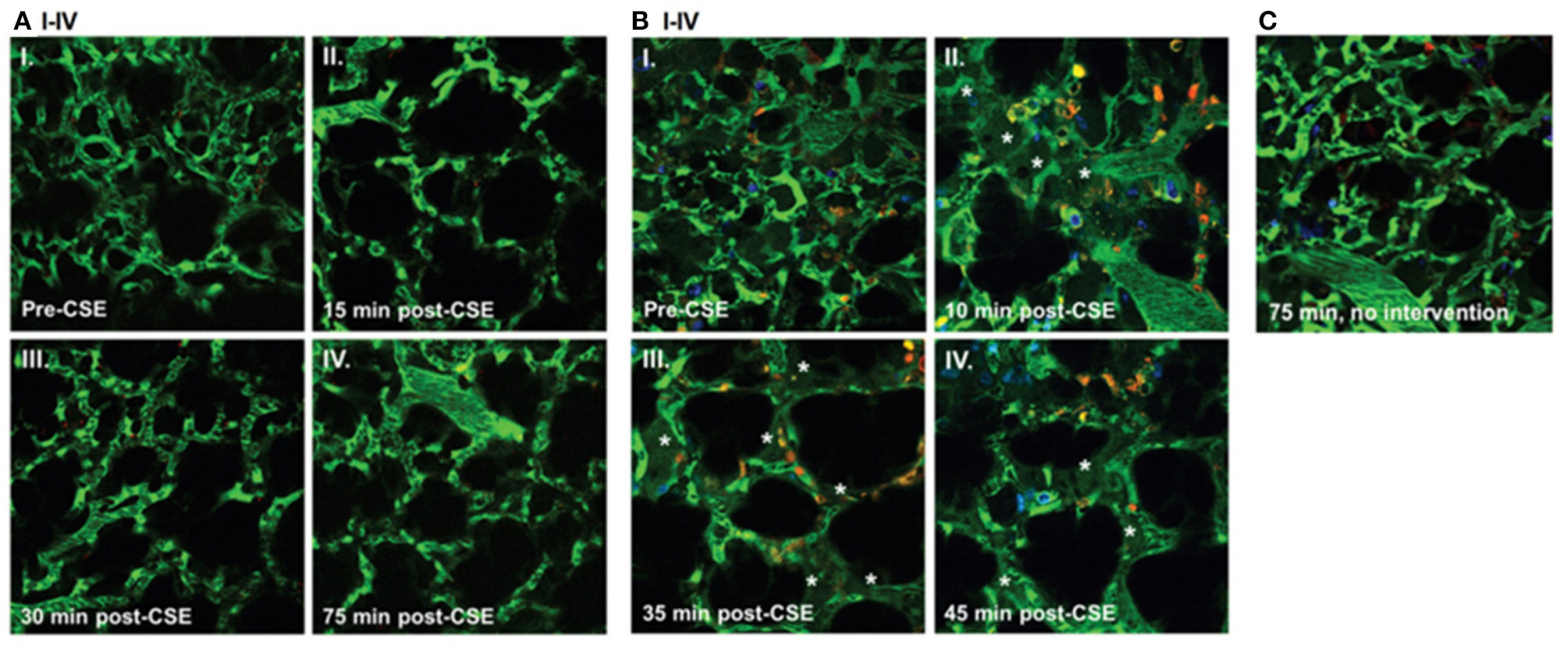
Effect of cigarette smoke extract (CSE) on the lung microcirculation captured in real time in the pulmonary microvasculature of a living wild-type (WT) **(A)**; and cystic fibrosis transmembrane conductance regulator (CFTR)–deficient **(B)** mouse. Three-dimensional reconstruction of fluorescein isothiocyanate– labeled vessels (green) surrounding alveoli (dark regions) and Rho-G6-labeled neutrophils (orange) imaged via intravital 2-photon microscopy before (**A**I and **B**I) and after (**A** and **B**II–IV) intravenous administration of CSE (100 μL of 20% CSE). Nuclei were stained with intravenous Hoechst (blue). Note increasing neutrophil trafficking and plasma extravasation (asterisks) into airspaces after CSE administration in the CFTR-deficient **(B)** but not WT **(A)** mouse and compared to a CFTR-deficient mouse not receiving CSE **(C)** (Brown et al., 2014).

Cystic Fibrosis (CF), an autosomal-recessive genetic disorder, causes chronic changes including inflammation of the lungs and other organs due to the defect in the cystic fibrosis transmembrane conductance regulator (CFTR). CF patients have a high incidence of lung infections limiting their quality of life. In this regard, there is only one study using intravital microscopy in CFTR mice thus far. Brown et al. studied the role of CFTR in preserving the lung endothelial barrier through the use of multiphoton microscopy. Thoracotomy was performed on the left chest wall to create a window for lung IVM. FITC-dextran (for labeling the circulating plasma), Hoechst 33258 (for labeling the nuclei), and Rhodamine-6G (for labeling of leukocyte mitochondria) were injected intravenously. Lung imaging in CFTR-deficient mice demonstrated a significant increase in neutrophil trafficking and plasma extravasation into airspaces following administration of cigarette smoke (CS) extracts compared to wild type mice exposed to similar level of CS extracts (see Figure 2). These results suggested that CFTR function might be required for lung endothelial barrier, including adherence junction stability. Loss of CFTR function, especially concomitant to CS exposure, might promote lung inflammation by increasing endothelial cell permeability. The results from *in vitro* experiments in this study also indicated that dose-dependent treatment with CFTR inhibitors such as GlyH-101 or CFTRinh172 was associated primarily with the redistribution of the junctional protein ß-catenin and its internalization from cell periphery. CS had inhibitory effects on CFTR function. It caused sphingosine-1 phosphate (S1P)/ceremide imbalance in which the enhanced level of ceremide leads to lung inflammation and increased susceptibility to *P. aeruginosa* infection. This imbalance and subsequent susceptibility to infection are also typical in clinical CF. S1P supplementation was able to reverse endothelial cell barrier dysfunction induced by CFTR inhibition or CS exposure (Brown et al., 2014). Furthermore, in *ex vivo* lung preparations, Lindert et al. studied alveoli from the pleural aspect down to a depth of up to 40 μm, using multiphoton microscopy to examine alveolar wall liquid (AWL). They found that AWL was absent in CFTR ^−/−^ mice, and it was blocked when chloride was depleted from the perfusate of WT mice, suggesting that CFTR-dependent chloride secretion causes AWL formation (Lindert et al., 2007).

### Sepsis Induced Acute Lung Injury

According to the third international consensus definitions for sepsis and septic shock (Sepsis-3), sepsis is a dysregulated host systemic responses to infection, that can cause life-threatening organ dysfunction (Singer et al., 2016). Sepsis affects more than 30 million people every year worldwide, and is one of the major causes of death (Vincent, 2012; Fleischmann et al., 2016). Therefore, understanding the pathogenesis of sepsis is a critical step in early diagnosis and treatment of sepsis which can limit onset of organ dysfunction and reduce mortality (Kim and Choi, 2020). The various aspects of sepsis pathogenesis include immune dysfunction, endothelial activation, cardiopulmonary pathology, increased microvascular permeability, edema formation, and disseminated intravascular coagulation (DIC) (Aird, 2003; Gotts and Matthay, 2016). Infection sites in septic patients include the abdomen, bloodstream, lung, central nervous system, and renal or genitourinary tract (Gotts and Matthay, 2016). Alternatively, sepsis also has the potential to induce acute lung injury (ALI) without primary lung infection. Lung IVM represents a useful method for studying pulmonary microcirculation changes in sepsis-induced ALI. The following paragraphs summarize various studies on sepsis-induced ALI and describe application of lung IVM in septic mice models.

Yipp et al. assessed the roles of CD11b, TLR4, and Myd88 in pulmonary neutrophil host defense during sepsis through the use of knockout mice. Researchers applied a vacuum chamber on the exposed left lung to facilitate pulmonary imaging by means of either spinning disk or resonant scanning confocal microscopy. Ten minutes prior to imaging, fluorescence-conjugated anti-Ly6G antibody and anti-CD31 antibody were administered intravenously in order to visualize neutrophils and the vascular endothelium. Three behavioral phenotypes of neutrophils were directly visualized within the pulmonary microvasculature during sepsis: tethering, crawling, and adhering. Results indicated no significant changes in the number of crawling neutrophils within pulmonary capillaries following administration of intravenous LPS in CD11b-, TLR4-, and Myd88-knockout mice, thereby confirming the role of these molecules in pulmonary neutrophil host defense (Yipp et al., 2017).

To demonstrate apoptosis of pulmonary microvascular endothelial cells (PMVEC) in sepsis-induced ALI, Gill et al. conducted lung IVM (Gill et al., 2014, 2015) in anesthetized, tracheotomized, mechanically ventilated mice. A transparent window on the right thoracic wall allowed visualization of the pulmonary microcirculation with an epi-fluorescence microscope (Razavi et al., 2004). A bolus of Rhodamine 6G was injected into the penile vein 3.5 h after sham or CLP surgery to label pulmonary microvascular PMN sequestration (Gill et al., 2014). Propidium iodide (PI), a fluorescent marker of cell death used to label non-viable PMVEC (Gill et al., 2014, 2015), was intravenously injected into septic mice immediately before IVM. Quantification of the number of PI positive cells and Rhodamine 6G labeled PMN sequestrated in recorded images indicated a significant increase in PMN sequestrated in pulmonary microvasculature. However, it is unclear whether the authors employed PI and Rhodamine 6G in separate experiments or in the same animals, which would raise the issue of separating fluorophores with such close emission wavelengths. Nonetheless, the authors' findings are consistent with results from other studies (Razavi et al., 2004; Roller et al., 2013) and with increased PMVEC death following CLP-induced sepsis at 2 and 4 h after CLP-sepsis compared to sham (Gill et al., 2014, 2015).

Razavi et al. quantified pulmonary microvascular neutrophil sequestration by IVM with intravenous injection of dihydro-rhodamine-6G. Results indicated that pulmonary microvascular leukocyte sequestration was significantly increased in iNOS+/+ (wild-type) mice and in iNOS^−/−^ mice from 1 to 18 h after CLP, although significantly fewer sequestered leukocytes were observed in iNOS^−/−^ vs. iNOS^+/+^ mice at all time points (Razavi et al., 2004). Therefore, neutrophil iNOS appears to be an important contributor to pulmonary neutrophil infiltration.

**Figure 3:**
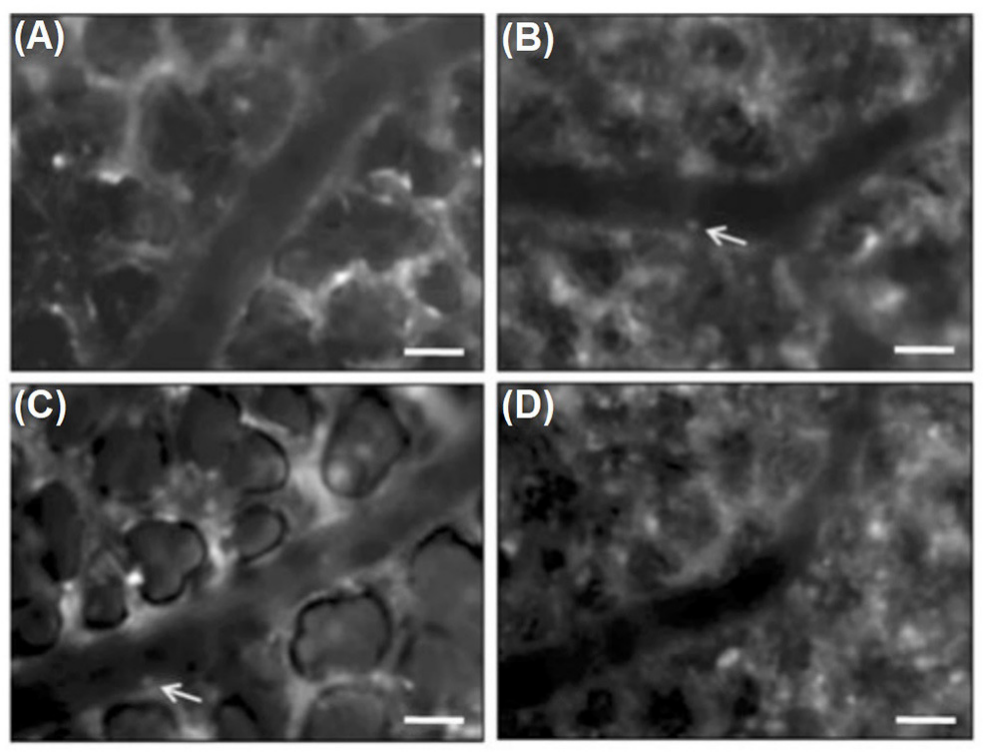
Intravital fluorescence microscopy of adherent leukocytes (arrows) in pulmonary arterioles. Note, that CLP enhanced the number of firmly adherent leukocytes in arterioles by two-fold (**B**, arrows) compared to sham-treated animals (**A**, arrows). Immunoneutralization of PSGL-1 reduced CLP-induced leukocyte adhesion in pulmonary arterioles by almost 50 % **(C,D)**. Green-light epi-illumination with direct staining of leukocytes by rhodamine 6 G. Scale bars 35 lm (Roller et al., 2013).

Rahman et al. used a micromanipulator to fix the coverslip to the right lung surface during surgical preparation. After retrobulbar injection of rhodamine 6G and FITC dextran, fluorescence microscopy was performed. Results indicated that the matrix metalloproteinase (MMP) inhibitor, GM6001, administered prior to CLP induction reduced CLP-induced leukocyte adhesion in pulmonary venules, which suggests the role of metalloproteinases in infiltration of neutrophils in septic lung injury (Rahman et al., 2012). Another study from this group (Roller et al., 2013) with the equal intravital microscopic setup demonstrated significant increases in the number of rolling and adhering leukocytes in arterioles and venules, as well as in leukocytes trapped in capillaries, 4 h after CLP induction (see Figure 3). IVM indicated that CLP induction markedly decreased flow velocity and shear rate in pulmonary venules and arterioles and also decreased functional capillary density in lung microcirculation (Roller et al., 2013).

Lung IVM was also used to show that immunoneutralization of PSGL-1 significantly reduces not only the number of rolling leukocytes in arterioles and venules but also the number of adherent leukocytes in arterioles and trapped leukocytes in capillaries. In one particular study, PSGL-1 antibody was shown not to affect the number of firm leukocytes in pulmonary venules, thus suggesting that leukocyte rolling is not a prerequisite for pulmonary venular leukocytes adhesion in sepsis. In addition, capillary trapping of leukocytes and enhanced sticky leukocytes in arterioles is dependent on PSGL-1 function (Roller et al., 2013).

Moreover, several studies have shown that targeting CD11a/CD18 or CD11b/CD18 reduces infiltration of leukocytes in the lung in models of endotoxemia (Basit et al., 2006) and sepsis (Asaduzzaman et al., 2008). To confirm this theory with *in vivo* imaging, Wang et al. performed lung IVM 4 h after CLP induction. Results indicated that intravenous pretreatment with CD11a or CD11b antibodies immediately prior to CLP abolished CLP-induced arteriolar and venular leukocyte adhesion in lung tissue and reduced leukocyte sequestration in pulmonary capillaries. It also restored diameter, flow velocity, and shear rate in lung arterioles and venules in CLP mice. Their findings showed that CD11a and CD11b mediate leukocyte adhesion in both arterioles and venules as well as trapping in capillaries in the lung. In addition, the data demonstrates that CD11a, but not CD11b, supports leukocyte rolling in pulmonary arterioles (Wang et al., 2013).

### Aspiration Pneumonia

Intratracheal instillation of hydrochloric acid is an experimental mouse model that mimics human aspiration of gastric contents, which can cause acute ALI (Kobayashi et al., 2016). Aspiration is classified under the non-infectious group of ALI etiology (Zarbock and Ley, 2009). Acid aspiration causes direct injury to lung epithelial and endothelial cells, leading to tissue edema and neutrophil accumulation in the lung (Kobayashi et al., 2016).

Grommes et al. performed lung IVM in mice after acid aspiration. Microspheres coupled to polyclonal antibodies to CXCL4 or CCL5 were injected intravenously 15 min prior to intravital imaging using a multiphoton system in single-beam mode. The authors showed the deposition of CCL5 and CXCL4 on microvascular lung endothelium and reported that platelets in LPS-, acid-, and sepsis-induced ALI release the CCL5-CXCL4 heterodimer. This heterodimer is involved in neutrophil recruitment, and its disruption prevents acid- and sepsis induced ALI (Grommes et al., 2012). Additionally, it has been shown that thromboxane A2 (TXA_2_), which is actively involved in the inflammatory response, is produced by lung epithelial cells in aggregation with platelets and neutrophils (Zarbock and Ley, 2009) and is detectable in bronchoalveolar fluids (BALF) in acid aspiration-induced ALI (Kobayashi et al., 2016). TXA_2_ binding to its receptor on the epithelial cells promotes upregulation of intracellular adhesion molecule-1 (ICAM-1) and may be indirectly involved in neutrophil recruitment (Rossaint and Zarbock, 2013).

Mertens et al. implemented Tabuchi et al. method (Tabuchi et al., 2008) for thoracic window implantation to analyze dynamics of alveolar clusters at different time points and applied pressures after HCl aspiration. Mice were ventilated at 60 breaths/ min and images were captured at 0 cm H2O ventilation pressure in end-expiration and at 6, 12, 18, 24 cm H2O in end-inspiration. Subpleural alveoli were visualized using an upright microscope and darkfield illumination, 30 min after lung stabilization. Delimited aerated structures discernible on the lung surface were defined as individual alveolar clusters. For each pressure step, in each area of interest (AOI), the number of alveolar clusters was counted and the boundaries of the subpleural projection of each cluster were traced, and the respective area was measured. Alveolar compliance was calculated as the fold increase in alveolar area between images taken at 0 and 24 cm H2O ventilation pressure. The total number of visualized alveolar cluster per AOI, after acid aspiration, did not change between 0 and 24 cm H2O. Dark-field illumination also demonstrated that density of light-refracting structures in the acid aspiration group increased. In the NaCl instillation (control) group, these structures disappeared with increasing ventilatory pressure, while in the HCl instillation group, they no longer disappeared with alveolar expansion. Additionally, alveolar distensibility was primarily reduced in small alveoli in acid-induced ALI. Optical Coherence Tomography (OCT) imaging also showed increased heterogeneous density in acid-injured lungs to confirm the IVM results (Mertens et al., 2009). In healthy lungs, cyclic changes in alveolar size are in synchrony with the ventilatory cycle, whereas asynchronous alveolar dynamics occurs in 10 min after ALI induced by acid instillation (Tabuchi et al., 2016). Altered alveolar dynamics in ALI cause impaired respiratory mechanics and alveolar gas exchange. Hence, requiring high distending pressures between and within alveoli results in the progression and aggravation of lung disease (Mertens et al., 2009; Tabuchi et al., 2016; Mandler et al., 2018).

## Limitations

Although lung IVM provides unique insights into pulmonary microvascular responses, cell-cell-interactions as well as alveolar mechanics in real time, it also carries some limitations due to the enclosed position of the lung in the body and restricted accessibility of the organ, thus necessitating the implementation of a surgical window for imaging. In some studies, part of the thoracic wall was removed to provide access for lung *in vivo* imaging without providing any protection for the parenchyma. However, this area of observation is prone to drying and is exposed to environmental pathogens and/or artificial surfaces (including cover slips) in these approaches. Moreover, cyclic lung movement during respiration and the heartbeat's effect on the lung both impact lung imaging due to motion artifacts. Some lung windows, for example, employ a thoracic suction window fitted with a vacuum chamber, and are implanted to stabilize the lung surface for improved imaging with reduced motion artifacts (Looney et al., 2011). While this technique has proven highly efficient for immunology studies, it does disrupt certain physiological phenomena such as ventilation-dependent effects on lung perfusion and alveolar dynamics. Some lung windows employ glued coverslips (Kreisel et al., 2010) or transparent polyvinylidene membranes (Tabuchi et al., 2008) instead of suction and allow physiological movement of the lung, but do not provide high resolution imaging.

Another factor that should be taken into consideration is the physiologically negative pressure in the thoracic cavity. This necessitates the use of great caution during the surgery as well as application of ventilatory support to keep the animal alive. Therefore, imaging must be performed under positive pressure ventilation instead of spontaneous breathing (Looney et al., 2011). However, the effects of artificial ventilation on lung physiology must be acknowledged (Poobalasingam et al., 2017). Manipulations in mechanics of the thoracic wall and the interplay between intrapleural and transpulmonary pressures impact both alveolar dynamics and microvascular perfusion (Tabuchi et al., 2020). Therefore, although mechanical ventilation is essential for survival of the animal, it can cause permanent damage to the lung as ventilator induced lung injury (VILI) or ventilator-associated lung injury (VALI) (Amato et al., 1998).

Furthermore, application of IVM in longitudinal studies has been faced with limitations as most implanted lung windows are too invasive to allow long term imaging. Since lung IVM is a terminal method used for short term imaging, multiple animals are needed for longer studies (Masterson et al., 2019). However, it has been reported that certain lung windows may allow for long-term observations in dogs, rabbits (De Alva and Rainer, 1963), rats (Fingar et al., 1994) or mice (Kimura et al., 2010), without stabilization and at low spatial resolution. Entenberg et al. recently developed a permanent lung window which may present a promising method for long-term imaging (Entenberg et al., 2018; Tabuchi et al., 2020).

There are some limitations that apply to all models and methods of lung IVM in mice regardless of different adaptions based on the subject of study. First, the small size of mice means that miniature equipment is required for IVM, thus making intubation or tracheotomy challenging. A typical mouse lung window is ~10 mm in diameter and provides a very small observational area during imaging (Masterson et al., 2019). Stabilizing the lung via thoracic chamber implantation is also a challenge in mice as a result of their smaller size and higher respiratory rate in comparison to larger animals (Frevert et al., 2014). Second, although classical fluorescence IVM provides adequate spatial resolution, its two-dimensional imaging provides little information about three-dimensional alveolar dynamics such as alveolar walls and alveolar diameter during different physiological and pathophysiological conditions (Tabuchi et al., 2020). Third, limited penetration depth of current microscopes allows only for superficial imaging of the lung. Penetration depth in lung tissue for conventional fluorescence microscopy is ~30–50 μm, compared to 100–150 μm for confocal microscopy, and 500 μm for multiphoton microscopy. This restricted penetration depth is caused by the lung's complex structure and the light-scattering properties of the abundant air-liquid interfaces in the alveolo-capillary units. Therefore, lung IVM is currently restricted to superficial layers of the lung including subpleural alveoli and surface-level microvascular networks, neither of which are representative of internal alveoli and vessels. In contrast to deeper lung regions in which all sides of a given alveolus are surrounded by other alveoli, subpleural alveoli are attached to the visceral pleura on one side. Consequently, alveolar dynamics differ between subpleural alveoli and deeper lung regions. In addition, subpleural pulmonary microvasculature is less dense and consists of larger capillaries compared to the interior pulmonary microvascular network (Tabuchi et al., 2008, 2020; Kuebler, 2011; Looney et al., 2011; Looney and Bhattacharya, 2014; Masterson et al., 2019). Fourth, creating a window to access the lung involves a surgical procedure which can cause baseline leukocyte activation. However, these alterations in leukocyte behavior are not comparable to inflammatory situations such as response to bacterial infection. Hence, exercising extreme care during surgery and comparing *in vivo* images with histological figures could lessen this issue (Hickey and Westhorpe, 2013). Fifth, lung observation by means of IVM is limited to a specific location that is not typically representative of the whole lung, usually the anterior part of the murine lung and a specific number of alveoli and microvessels within this area. It should be noted that ventilation and perfusion vary between different regions of the lung. Furthermore, any IVM–not only of the lungs–has to be performed at physiological body temperature, which in the case of mice is 37°C, by use of a homeothermic system (Park et al., 2018), warmed lung window chambers (Kreisel et al., 2010), or water immersion microscopy and continuous superfusion of the window with a pre-warmed solution (Kuhnle et al., 1993; Kuebler et al., 1994). IVM cannot be applied in clinical studies as it is an invasive and terminal method. Although some labels used in intravital imaging are non-toxic, do not alter cell morphology or phenotype, and have a sensitivity at micron range, they likely compromise either the administered cell's function or the host itself.

## Conclusion

Despite the aforementioned limitations, lung IVM is the gold standard for studying lung immune cell interactions in real time in living animals. This technique can be applied in different inflammatory models, including bacterial and viral infections, sepsis-induced ALI, aspiration, and cystic fibrosis in order to reveal dynamic alterations in physiological parameters and cellular behavior in comparison to non-inflammatory conditions.

## Author Contributions

All authors listed have made a substantial, direct and intellectual contribution to the work, and approved it for publication.

## Conflict of Interest

The authors declare that the research was conducted in the absence of any commercial or financial relationships that could be construed as a potential conflict of interest.

## References

[B1] Aderem A. Ulevitch R. J. (2000). Toll-like receptors in the induction of the innate immune response. Nature 406:782. 10.1038/35021228 10963608

[B2] Aird W. C. (2003). The role of the endothelium in severe sepsis and multiple organ dysfunction syndrome. Blood J. Am. Soc. Hematol. 101, 3765–3777. 10.1182/blood-2002-06-1887 12543869

[B3] Alves N. G. Motawe Z. Y. Yuan S. Y. Breslin J. W. (2018). Endothelial protrusions in junctional integrity and barrier function. Curr. Top. Membr. 82, 93–140. 10.1016/bs.ctm.2018.08.006 30360784 PMC6442684

[B4] Amato M. B. P. Barbas C. S. V. Medeiros D. M. Magaldi R. B. Schettino G. P. Lorenzi-Filho G.. (1998). Effect of a protective-ventilation strategy on mortality in the acute respiratory distress syndrome. N. Engl. J. Med. 338, 347–354. 10.1056/NEJM199802053380602 9449727

[B5] Asaduzzaman M. Zhang S. Lavasani S. Wang Y. Thorlacius H. (2008). LFA-1 and MAC-1 mediate pulmonary recruitment of neutrophils and tissue damage in abdominal sepsis. Shock 30, 254–259. 10.1097/shk.0b013e318162c567 18197144

[B6] Basit A. Reutershan J. Morris M. A. Solga M. Rose C. E. Jr. Ley K. (2006). ICAM-1 and LFA-1 play critical roles in LPS-induced neutrophil recruitment into the alveolar space. Am. J. Physiol. Cell Mol. Physiol. 291, L200–L207. 10.1152/ajplung.00346.2005 16461431

[B7] Blank F. Wehrli M. Lehmann A. Baum O. Gehr P. von Garnier C.. (2011). Macrophages and dendritic cells express tight junction proteins and exchange particles in an *in vitro* model of the human airway wall. Immunobiology 216, 86–95. 10.1016/j.imbio.2010.02.006 20362352

[B8] Broide D. H. Humber D. Sriramarao P. (1998). Inhibition of eosinophil rolling and recruitment in P-selectin–and intracellular adhesion molecule-1–deficient mice. Blood 91, 2847–2856. 10.1182/blood.V91.8.2847.2847_2847_2856 9531595

[B9] Brown M. B. Hunt W. R. Noe J. E. Rush N. I. Schweitzer K. S. Leece T. C.. (2014). Loss of cystic fibrosis transmembrane conductance regulator impairs lung endothelial cell barrier function and increases susceptibility to microvascular damage from cigarette smoke. Pulm Circ. 4, 260–268. 10.1086/675989 25006445 PMC4070785

[B10] Carestia A. Davis R. P. Davis L. Jenne C. N. (2019). Inhibition of immunothrombosis does not affect pathogen capture and does not promote bacterial dissemination in a mouse model of sepsis. Platelets. 31, 925–931. 10.1080/09537104.2019.1704711 31851856

[B11] Chiang E. Y. Hidalgo A. Chang J. Frenette P. S. (2007). Imaging receptor microdomains on leukocyte subsets in live mice. Nat. Methods 4:219. 10.1038/nmeth1018 17322889

[B12] Chtanova T. Schaeffer M. Han S.-J. van Dooren G. G. Nollmann M. Herzmark P.. (2008). Dynamics of neutrophil migration in lymph nodes during infection. Immunity 29, 487–496. 10.1016/j.immuni.2008.07.012 18718768 PMC2569002

[B13] Cleret A. Quesnel-Hellmann A. Vallon-Eberhard A. Verrier B. Jung S. Vidal D. (2007). Lung dendritic cells rapidly mediate anthrax spore entry through the pulmonary route. J. Immunol. 178, 7994–8001. 10.4049/jimmunol.178.12.7994 17548636

[B14] Croix C. M. S. Leelavanichkul K. Watkins S. C. (2006). Intravital fluorescence microscopy in pulmonary research. Adv. Drug Deliv. Rev. 58, 834–840. 10.1016/j.addr.2006.07.007 16996641

[B15] De Alva W. E. Rainer W. G. (1963). A method of high speed *in vivo* pulmonary microcinematography under physiologic conditions. Angiology 14, 160–164. 10.1177/000331976301400402 14025662

[B16] Entenberg D. Rodriguez-Tirado C. Kato Y. Kitamura T. Pollard J. W. Condeelis J. (2015). *In vivo* subcellular resolution optical imaging in the lung reveals early metastatic proliferation and motility. Intravital 4, 1–11. 10.1080/21659087.2015.1086613 26855844 PMC4737962

[B17] Entenberg D. Voiculescu S. Guo P. Borriello L. Wang Y. Karagiannis G. S.. (2018). A permanent window for the murine lung enables high-resolution imaging of cancer metastasis. Nat. Methods 15:73. 10.1038/nmeth.4511 29176592 PMC5755704

[B18] Faust N. Varas F. Kelly L. M. Heck S. Graf T. (2000). Insertion of enhanced green fluorescent protein into the lysozyme gene creates mice with green fluorescent granulocytes and macrophages. Blood 96, 719–726. 10.1182/blood.V96.2.719 10887140

[B19] Fingar V. H. Taber S. W. Wieman T. J. (1994). A new model for the study of pulmonary microcirculation: determination of pulmonary edema in rats. J. Surg. Res. 57, 385–393. 10.1006/jsre.1994.1159 8072287

[B20] Fiole D. Deman P. Trescos Y. Mayol J.-F. Mathieu J. Vial J.-C.. (2014). Two-photon intravital imaging of lungs during anthrax infection reveals long-lasting macrophage-dendritic cell contacts. Infect. Immun. 82, 864–872. 10.1128/IAI.01184-13 24478099 PMC3911401

[B21] Fiole D. Tournier J. (2016). Intravital microscopy of the lung: minimizing invasiveness. J. Biophotonics 9, 868–878. 10.1002/jbio.201500246 26846880

[B22] Fleischmann C. Scherag A. Adhikari N. K. J. Hartog C. S. Tsaganos T. Schlattmann P.. (2016). Assessment of global incidence and mortality of hospital-treated sepsis. Current estimates and limitations. Am. J. Respir. Crit. Care Med. 193, 259–272. 10.1164/rccm.201504-0781OC 26414292

[B23] Fournier B. Philpott D. J. (2005). Recognition of *Staphylococcus aureus* by the innate immune system. Clin. Microbiol. Rev. 18, 521–540. 10.1128/CMR.18.3.521-540.2005 16020688 PMC1195972

[B24] Frevert U. Nacer A. Cabrera M. Movila A. Leberl M. (2014). Imaging Plasmodium immunobiology in the liver, brain, and lung. Parasitol. Int. 63, 171–186. 10.1016/j.parint.2013.09.013 24076429 PMC3876283

[B25] Funakoshi N. Onizuka M. Yanagi K. Ohshima N. Tomoyasu M. Sato Y.. (2000). A new model of lung metastasis for intravital studies. Microvasc. Res. 59, 361–367. 10.1006/mvre.2000.2238 10792967

[B26] Garcia J. A. Cardona S. M. Cardona A. E. (2013). Analyses of microglia effector function using CX3CR1-GFP knock-in mice, in Microglia. Methods in Molecular Biology (Methods and Protocols), Vol. 1041, eds B. Joseph and J. Venero (Totowa, NJ: Humana Press), 307–317. 10.1007/978-1-62703-520-0_27 PMC3980416 23813389

[B27] Gill S. E. Rohan M. Mehta S. (2015). Role of pulmonary microvascular endothelial cell apoptosis in murine sepsis-induced lung injury *in vivo*. Respir. Res. 16:109. 10.1186/s12931-015-0266-7 26376777 PMC4574190

[B28] Gill S. E. Taneja R. Rohan M. Wang L. Mehta S. (2014). Pulmonary microvascular albumin leak is associated with endothelial cell death in murine sepsis-induced lung injury *in vivo*. PLoS ONE 9:e88501. 10.1371/journal.pone.0088501 24516666 PMC3917898

[B29] Grommes J. Alard J.-E. Drechsler M. Wantha S. Mörgelin M. Kuebler W. M.. (2012). Disruption of platelet-derived chemokine heteromers prevents neutrophil extravasation in acute lung injury. Am. J. Respir. Cri.t Care Med. 185, 628–636. 10.1164/rccm.201108-1533OC 22246174 PMC3326286

[B30] Grott K. Dunlap J. D. (2016). Atelectasis, in StatPearls (Treasure Island, FL: StatPearls Publishing).

[B31] Grott K. Dunlap J. D. (2020). Atelectasis. StatPearls.31424900

[B32] Herman B. (1998). Fluorescence microscopy, in Fluorescence Microscopy (New York, NY: Springer-Verlag), 15–38.

[B33] Hickey M. J. Westhorpe C. L. V. (2013). Imaging inflammatory leukocyte recruitment in kidney, lung and liver—challenges to the multi-step paradigm. Immunol. Cell Biol. 91, 281–289. 10.1038/icb.2012.83 23337698

[B34] Khandoga A. Kessler J. S. Meissner H. Hanschen M. Corada M. Motoike T.. (2005). Junctional adhesion molecule-A deficiency increases hepatic ischemia-reperfusion injury despite reduction of neutrophil transendothelial migration. Blood 106, 725–733. 10.1182/blood-2004-11-4416 15827135

[B35] Kim H. K. Missiakas D. Schneewind O. (2014). Mouse models for infectious diseases caused by *Staphylococcus aureus*. J. Immunol. Methods 410, 88–99. 10.1016/j.jim.2014.04.007 24769066 PMC6211302

[B36] Kim M.-H. Choi J.-H. (2020). An update on sepsis biomarkers. Infect. Chemother. 52, 1–18. 10.3947/ic.2020.52.1.1 32239808 PMC7113456

[B37] Kim Y. M. Jeong S. Choe Y. H. Hyun Y.-M. (2019). Two-photon intravital imaging of leukocyte migration during inflammation in the respiratory system. Acute Crit Care 34, 101–107. 10.4266/acc.2019.00542 31723914 PMC6786666

[B38] Kimura H. Hayashi K. Yamauchi K. Yamamoto N. Tsuchiya H. Tomita K.. (2010). Real-time imaging of single cancer-cell dynamics of lung metastasis. J. Cell Biochem. 109, 58–64. 10.1002/jcb.22379 19911396

[B39] Kobayashi K. Horikami D. Omori K. Nakamura T. Yamazaki A. Maeda S.. (2016). Thromboxane A 2 exacerbates acute lung injury via promoting edema formation. Sci. Rep. 6:32109. 10.1038/srep32109 27562142 PMC4999811

[B40] Krahl V. E. (1963). A method of studying the living lung in the closed thorax, and some preliminary observations. Angiology 14, 149–159. 10.1177/000331976301400401 14035399

[B41] Kramer K. Voss H.-P. Grimbergen J. A. Mills P. A. Huetteman D. Zwiers L.. (2000). Telemetric monitoring of blood pressure in freely moving mice: a preliminary study. Lab. Anim. 34, 272–280. 10.1258/002367700780384663 11037121

[B42] Kreisel D. Nava R. G. Li W. Zinselmeyer B. H. Wang B. Lai J.. (2010). In vivo two-photon imaging reveals monocyte-dependent neutrophil extravasation during pulmonary inflammation. Proc. Natl. Acad. Sci.U.S.A. 107, 18073–18078. 10.1073/pnas.1008737107 20923880 PMC2964224

[B43] Kuebler W. M. (2011). Real-time imaging assessment of pulmonary vascular responses. Proc. Am. Thorac. Soc. 8, 458–465. 10.1513/pats.201101-005MW 22052920

[B44] Kuebler W. M. Kuhnle G. E. Groh J. Goetz A. E. (1994). Leukocyte kinetics in pulmonary microcirculation: intravital fluorescence microscopic study. J. Appl. Physiol. 76, 65–71. 10.1152/jappl.1994.76.1.65 8175549

[B45] Kuebler W. M. Parthasarathi K. Lindert J. Bhattacharya J. (2007). Real-time lung microscopy. J. Appl. Physiol. 102, 1255–1264. 10.1152/japplphysiol.00786.2006 17095639

[B46] Kuhnle G. E. Leipfinger F. H. Goetz A. E. (1993). Measurement of microhemodynamics in the ventilated rabbit lung by intravital fluorescence microscopy. J. Appl. Physiol. 74, 1462–1471. 10.1152/jappl.1993.74.3.1462 8482691

[B47] Kukavica-Ibrulj I. Bragonzi A. Paroni M. Winstanley C. Sanschagrin F. O'Toole G. A. (2008). In vivo growth of *Pseudomonas aeruginosa* strains PAO1 and PA14 and the hypervirulent strain LESB58 in a rat model of chronic lung infection. J. Bacteriol. 190, 2804–2813. 10.1128/JB.01572-07 18083816 PMC2293253

[B48] Lavoie E. G. Wangdi T. Kazmierczak B. I. (2011). Innate immune responses to *Pseudomonas aeruginosa* infection. Microbes Infect. 13, 1133–1145. 10.1016/j.micinf.2011.07.011 21839853 PMC3221798

[B49] Lee P. Y. Wang J. Parisini E. Dascher C. C. Nigrovic P. A. (2013). Ly6 family proteins in neutrophil biology. J. Leukoc Biol. 94, 585–594. 10.1189/jlb.0113014 23543767

[B50] Lefrançais E. Mallavia B. Looney M. R. (2017). Lung imaging in animal models, in Acute Lung Injury and Repair. (Springer), 107–32. 10.1007/978-3-319-46527-2_8

[B51] Lefrançais E. Mallavia B. Zhuo H. Calfee C. S. Looney M. R. (2018). Maladaptive role of neutrophil extracellular traps in pathogen-induced lung injury. JCI Insight 3:e98178. 10.1172/jci.insight.98178 29415887 PMC5821185

[B52] Lichtman J. W. Conchello J.-A. (2005). Fluorescence microscopy. Nat. Methods 2, 910–919. 10.1038/nmeth817 16299476

[B53] Lien D. C. Wagner W. W. Jr. Capen R. L. Haslett C. Hanson W. L. Hofmeister S. E.. (1987). Physiological neutrophil sequestration in the lung: visual evidence for localization in capillaries. J. Appl. Physiol. 62, 1236–1243. 10.1152/jappl.1987.62.3.1236 3106311

[B54] Lindert J. Perlman C. E. Parthasarathi K. Bhattacharya J. (2007). Chloride-dependent secretion of alveolar wall liquid determined by optical-sectioning microscopy. Am. J. Respir. Cell Mol. Biol. 36, 688–696. 10.1165/rcmb.2006-0347OC 17290033 PMC1899339

[B55] Lindon J. C. Tranter G. E. Koppenaal D. (2016). Encyclopedia of Spectroscopy and Spectrometry. Oxford, UK: Academic Press.

[B56] Looney M. R. Bhattacharya J. (2014). Live imaging of the lung. Annu. Rev. Physiol. 76, 431–445. 10.1146/annurev-physiol-021113-170331 24245941 PMC4501380

[B57] Looney M. R. Thornton E. E. Sen D. Lamm W. J. Glenny R. W. Krummel M. F. (2011). Stabilized imaging of immune surveillance in the mouse lung. Nat. Methods. 8:91. 10.1038/nmeth.1543 21151136 PMC3076005

[B58] Mandler W. K. Nurkiewicz T. R. Porter D. W. Kelley E. E. Olfert I. M. (2018). Microvascular dysfunction following multiwalled carbon nanotube exposure is mediated by Thrombospondin-1 receptor CD47. Toxicol. Sci. 165, 90–99. 10.1093/toxsci/kfy120 29788500 PMC6111784

[B59] Margraf A. Herter J. M. Kühne K. Stadtmann A. Ermert T. Wenk M. (2018). 6% Hydroxyethyl starch (HES 130/0.4) diminishes glycocalyx degradation and decreases vascular permeability during systemic and pulmonary inflammation in mice. Crit. Care 22:111 10.1186/s13054-017-1846-3 29716625 PMC5930811

[B60] Masedunskas A. Milberg O. Porat-Shliom N. Sramkova M. Wigand T. Amornphimoltham P.. (2012). Intravital microscopy: a practical guide on imaging intracellular structures in live animals. Bioarchitecture 2, 143–157. 10.4161/bioa.21758 22992750 PMC3696059

[B61] Masterson C. H. Curley G. F. Laffey J. G. (2019). Modulating the distribution and fate of exogenously delivered MSCs to enhance therapeutic potential: knowns and unknowns. Intensive Care Med. Exp. 7:41. 10.1186/s40635-019-0235-4 31346794 PMC6658643

[B62] McCormack D. G. Mehta S. Tyml K. Scott J. A. Potter R. Rohan M. (2000). Pulmonary microvascular changes during sepsis: evaluation using intravital videomicroscopy. Microvasc. Res. 60, 131–140. 10.1006/mvre.2000.2261 10964587

[B63] Medina-Contreras O. Geem D. Laur O. Williams I. R. Lira S. A. Nusrat A.. (2011). CX3CR1 regulates intestinal macrophage homeostasis, bacterial translocation, and colitogenic Th17 responses in mice. J. Clin. Invest. 121, 4787–4795. 10.1172/JCI59150 22045567 PMC3226003

[B64] Mempel T. R. Moser C. Hutter J. Kuebler W. M. Krombach F. (2003). Visualization of leukocyte transendothelial and interstitial migration using reflected light oblique transillumination in intravital video microscopy. J. Vasc. Res. 40, 435–441. 10.1159/000073902 14530600

[B65] Mertens M. Tabuchi A. Meissner S. Krueger A. Schirrmann K. Kertzscher U.. (2009). Alveolar dynamics in acute lung injury: heterogeneous distension rather than cyclic opening and collapse. Crit. Care Med. 37, 2604–2611. 10.1097/CCM.0b013e3181a5544d 19623041

[B66] Moradali M. F. Ghods S. Rehm B. H. A. (2017). *Pseudomonas aeruginosa* lifestyle: a paradigm for adaptation, survival, and persistence. Front. Cell Infect. Microbiol. 7:39. 10.3389/fcimb.2017.00039 28261568 PMC5310132

[B67] Naumenko V. Van S Dastidar H. Kim D.-S. Kim S.-J. Zeng Z.. (2018). Visualizing oncolytic virus-host interactions in live mice using intravital microscopy. Mol. Ther. 10, 14–27. 10.1016/j.omto.2018.06.001 30073187 PMC6070694

[B68] Neupane A. S. Willson M. Chojnacki A. K. Castanheira F. V. E. S. Morehouse C. Carestia A.. (2020). Patrolling alveolar macrophages conceal bacteria from the immune system to maintain homeostasis. Cell 183, 110–125. 10.1016/j.cell.2020.08.020 32888431

[B69] Niesner R. Andresen V. Neumann J. Spiecker H. Gunzer M. (2007). The power of single and multibeam two-photon microscopy for high-resolution and high-speed deep tissue and intravital imaging. Biophys. J. 93, 2519–2529. 10.1529/biophysj.106.102459 17557785 PMC1965440

[B70] Noda S. Asano Y. Nishimura S. Taniguchi T. Fujiu K. Manabe I.. (2014). Simultaneous downregulation of KLF5 and Fli1 is a key feature underlying systemic sclerosis. Nat. Commun. 5:5797. 10.1038/ncomms6797 25504335 PMC4268882

[B71] Norman K. (2005). Techniques: Intravital microscopy–a method for investigating disseminated intravascular coagulation? Trends Pharmacol. Sci. 26, 327–332. 10.1016/j.tips.2005.04.002 15925708

[B72] Ochi H. Iijima T. Ushiyama A. (2019). Intra-vital observation of lung water retention following intravenous injection of anti-MHC-class I (H-2K) monoclonal antibody in mice. In Vivo (Brooklyn) 33, 1477–1484. 10.21873/invivo.11627 31471395 PMC6755016

[B73] Opal S. M. (2010). Endotoxins and other sepsis triggers, in Endotoxemia and Endotoxin Shock. (Karger Publishers), 14–24. 10.1159/000315915 20519895

[B74] Orthgiess J. Gericke M. Immig K. Schulz A. Hirrlinger J. Bechmann I.. (2016). Neurons exhibit Lyz2 promoter activity *in vivo*: implications for using LysM-Cre mice in myeloid cell research. Eur. J. Immunol. 46, 1529–1532. 10.1002/eji.201546108 27062494

[B75] Park I. Choe K. Seo H. Hwang Y. Song E. Ahn J.. (2018). Intravital imaging of a pulmonary endothelial surface layer in a murine sepsis model. Biomed. Opt. Express 9, 2383–2393. 10.1364/BOE.9.002383 29760995 PMC5946796

[B76] Poobalasingam T. Salman D. Li H. Alçada J. Dean C.H. (2017) Imaging the lung: the old ways the new. Histol. Histopathol. 32, 325–337. 10.14670/HH-11-827 27624638

[B77] Presson R. G. Jr. Brown M. B. Fisher A. J. Sandoval R. M. Dunn K. W. Lorenz K. S.. (2011). Two-photon imaging within the murine thorax without respiratory and cardiac motion artifact. Am. J. Pathol. 179, 75–82. 10.1016/j.ajpath.2011.03.048 21703395 PMC3123791

[B78] Rahman M. Roller J. Zhang S. Syk I. Menger M. D. Jeppsson B.. (2012). Metalloproteinases regulate CD40L shedding from platelets and pulmonary recruitment of neutrophils in abdominal sepsis. Inflamm. Res. 61, 571–579. 10.1007/s00011-012-0446-6 22349180

[B79] Razavi H. M. Wang L. F. Weicker S. Rohan M. Law C. McCormack D. G.. (2004). Pulmonary neutrophil infiltration in murine sepsis: role of inducible nitric oxide synthase. Am. J. Respir. Crit. Care Med. 170, 227–233. 10.1164/rccm.200306-846OC 15059787

[B80] Reutershan J. Basit A. Galkina E. V. Ley K. (2005). Sequential recruitment of neutrophils into lung and bronchoalveolar lavage fluid in LPS-induced acute lung injury. Am. J. Physiol. Cell Mol. Physiol. 289, L807–L815. 10.1152/ajplung.00477.2004 15951336

[B81] Rodriguez-Tirado C. Kitamura T. Kato Y. Pollard J. W. Condeelis J. S. Entenberg D. (2016). Long-term high-resolution intravital microscopy in the lung with a vacuum stabilized imaging window. JoVE J. Vis. Exp. 116:e54603. 10.3791/54603 27768066 PMC5092167

[B82] Roller J. Wang Y. Rahman M. Schramm R. Laschke M. W. Menger M. D.. (2013). Direct *in vivo* observations of P-selectin glycoprotein ligand-1-mediated leukocyte–endothelial cell interactions in the pulmonary microvasculature in abdominal sepsis in mice. Inflamm. Res. 62, 275–282. 10.1007/s00011-012-0575-y 23178793

[B83] Rossaint J. Zarbock A. (2013). Tissue-specific neutrophil recruitment into the lung, liver, and kidney. J. Innate Immun. 5, 348–357. 10.1159/000345943 23257511 PMC6741449

[B84] Sanderson M. J. Smith I. Parker I. Bootman M. D. (2014). Fluorescence microscopy. Cold Spring Harb. Protoc. 2014:pdb-top071795. 10.1101/pdb.top071795 PMC4711767 25275114

[B85] Singer M. Deutschman C. S. Seymour C. W. Shankar-Hari M. Annane D. Bauer M. (2016). The third international consensus definitions for sepsis and septic shock (Sepsis-3). JAMA 315, 801–810. 10.1001/jama.2016.0287 26903338 PMC4968574

[B86] Tabuchi A. Matuszak J. Kuebler W. M. (2020). Ventilation and perfusion at the alveolar level: insights from lung intravital microscopy. Front. Physiol. 11:291. 10.3389/fphys.2020.00291 32308629 PMC7145899

[B87] Tabuchi A. Mertens M. Kuppe H. Pries A. R. Kuebler W. M. (2008). Intravital microscopy of the murine pulmonary microcirculation. J. Appl. Physiol. 104, 338–346. 10.1152/japplphysiol.00348.2007 18006870

[B88] Tabuchi A. Nickles H. T. Kim M. Semple J. W. Koch E. Brochard L.. (2016). Acute lung injury causes asynchronous alveolar ventilation that can be corrected by individual sighs. Am. J. Respir. Crit. Care Med. 193, 396–406. 10.1164/rccm.201505-0901OC 26513710

[B89] Tauer U. (2002). Advantages and risks of multiphoton microscopy in physiology. Exp. Physiol. 87, 709–714. 10.1113/eph8702464 12530403

[B90] Vincent J.-L. (2012). Increasing awareness of sepsis: World Sepsis Day. Crit Care 16:152. 10.1186/cc11511 22971299 PMC3682256

[B91] Wagner W. W. (1965). Microscopic observation of the lung *in vivo*. Vasc. Dis. 2, 229–241. 5866200

[B92] Wagner W. W. Jr. (1969). Pulmonary microcirculatory observations *in vivo* under physiological conditions. J. Appl. Physiol. 26, 375–377. 10.1152/jappl.1969.26.3.375 5773180

[B93] Wang Y. Roller J. Menger M. D. Thorlacius H. (2013). Sepsis-induced leukocyte adhesion in the pulmonary microvasculature *in vivo* is mediated by CD11a and CD11b. Eur. J. Pharmacol. 702, 135–141. 10.1016/j.ejphar.2013.01.024 23380685

[B94] Wang Z. (2016). Imaging nanotherapeutics in inflamed vasculature by intravital microscopy. Theranostics 6:2431. 10.7150/thno.16307 27877245 PMC5118605

[B95] Wearn J. T. Barr J. S. German W. J. (1926). The Behavior of the Arterioles and Capillaries of the Lung. Proc. Soc. Exp. Biol. Med. 24, 114–115. 10.3181/00379727-24-3250

[B96] Wells W. A. Thrall M. Sorokina A. Fine J. Krishnamurthy S. Haroon A.. (2018). *In vivo and ex vivo* microscopy: moving toward the integration of optical imaging technologies into pathology practice. Arch. Pathol. Lab. Med. 143, 288–298. 10.5858/arpa.2018-0298-RA 30525931

[B97] Witte S. (1992). Fluorescence microscopic techniques in intravital microvascular studies: plasma proteins and cells. Behring Inst. Mitt. 91, 210–229. 1524568

[B98] Wynn T. A. Chawla A. Pollard J. W. (2013). Macrophage biology in development, homeostasis and disease. Nature 496:445. 10.1038/nature12034 23619691 PMC3725458

[B99] Yipp B. G. Kim J. H. Lima R. Zbytnuik L. D. Petri B. Swanlund N.. (2017). The lung is a host defense niche for immediate neutrophil-mediated vascular protection. Sci. Immunol. 2:eaam8929. 10.1126/sciimmunol.aam8929 28626833 PMC5472445

[B100] Zarbock A. Ley K. (2009). The role of platelets in acute lung injury (ALI). Front. Biosci. J. Virtual Libr. 14:150. 10.2741/3236 19273059 PMC2745111

